# Engineered T cell therapy for the treatment of cardiac fibrosis during chronic phase of myocarditis

**DOI:** 10.7150/thno.116749

**Published:** 2026-01-01

**Authors:** Xiumeng Hua, Zhe Sun, Ziwei Liang, Yanhong Huang, Han Mo, Fei Dong, Shimin Mo, Xingyue Yang, Ningning Zhang, Xiao Chen, Shumin Liao, Zhen Qi, Rosanna Zhang, Shuge Guan, Liang Li, Yang Xu, Jiangping Song

**Affiliations:** 1 State Key Laboratory of Cardiovascular Disease, Fuwai Hospital, National Center for Cardiovascular Diseases, Chinese Academy of Medical Sciences and Peking Union Medical College, Beijing 100037, China.; 2 Shenzhen State Key Laboratory of Cardiovascular Disease, Fuwai Hospital Chinese Academy of Medical Sciences, Shenzhen 518038, China.; 3 Department of Human Cell Biology and Genetics, School of Medicine, Southern University of Science and Technology, Shenzhen, 518055, China.; 4 Department of Pharmacology, Joint Laboratory of Guangdong-Hong Kong Universities for Vascular Homeostasis and Diseases, School of Medicine, Southern University of Science and Technology, Shenzhen, 518055, China.; 5 Department of Cardiac Surgery, Fuwai Hospital, National Center for Cardiovascular Diseases, Chinese Academy of Medical Sciences and Peking Union Medical College, Beijing 100037, China.; 6 Beijing Key Laboratory of Preclinical Research and Evaluation for Cardiovascular Implant Materials, Fuwai Hospital, National Center for Cardiovascular Diseases, Chinese Academy of Medical Sciences and Peking Union Medical College, Beijing 100037, China.; 7 Department of Cardiac Surgery, Fuwai Yunnan Hospital, Chinese Academy of Medical Sciences, Affiliated Cardiovascular Hospital of Kunming Medical University 650102, Kunming, China.; 8 1 EvoLab, ACROBiosystems Inc, Beijing 100176, China.

**Keywords:** myocardial fibrosis, CAR-T, myocarditis, fibroblast activation protein-α

## Abstract

**Background**: Chronic myocarditis (CMYO) progresses to fibrosis and heart failure, yet no therapies effectively target fibrosis. Fibroblast activation protein (FAP) marks pathogenic myofibroblasts, but its therapeutic potential remains unexplored in inflammatory settings.

**Methods**: Using bulk/scRNA-seq of human myocarditis samples, we identified FAP as a fibrosis-specific marker. We engineered FAP-targeted CAR-T (FAP.CAR-T) cells and tested their efficacy in autoimmune (EAM) and viral (CVB3) myocarditis models. Human cardiac organoids (hCOs) treated with IL-17A modeled inflammatory fibrosis.

**Results**: FAP expression correlated with fibrosis severity in patients (r = 0.96, P = 0.0028). In EAM and CVB3 models, FAP.CAR-T cells reduced fibrosis by 65% and 55%, respectively (P < 0.001), restored ejection fraction to higher than 65%. hCOs treated with FAP.CAR-T cells showed 55% less fibrosis (P < 0.05). No toxicity was observed in healthy mice.

**Conclusions**: FAP.CAR-T cells eliminate fibrosis-driving myofibroblasts, reversing cardiac dysfunction in chronic myocarditis. This strategy, validated in human organoids, offers translatable immunotherapy for fibrosis-driven heart disease.

## Introduction

Chronic myocarditis (CMYO) progresses to irreversible fibrosis and heart failure, yet no therapies directly target fibrosis-driving myofibroblasts 1-3. Persistent inflammation activates cardiac fibroblasts into fibroblast activation protein positive (FAP^+^) myofibroblasts 4, which secrete excessive collagen and excessive extracellular matrix (ECM), stiffening the myocardium and impairing systolic function 5, 6. While angiotensin converting enzyme (ACE) inhibitors or angiotensin receptor blockers (ARBs) delay fibrosis, they fail to eliminate activated myofibroblasts, highlighting the need for precision therapies 7.

Chimeric antigen receptor T-cell (CAR-T) therapy, engineered to eliminate specific pathogenic cells, offers promise 8-11. Epstein et al. pioneered FAP-targeted CAR-Ts, reducing fibrosis in hypertensive heart disease 4, 12, 13. However, their model excluded inflammatory contexts (e.g., viral or autoimmune myocarditis), where immune cell and cytokine-rich microenvironments may alter FAP^+^ cell susceptibility. Thus, it remains unclear whether the activation of myofibroblasts during chronic myocarditis showed equivalent features to that during other pathological conditions and whether targeted therapy against these fibroblasts in models that are driven by pathogenic antigen-specific T cells could also alleviate cardiac dysfunction as in previous studies.

Here, we address these gaps by testing FAP.CAR-T cells in autoimmune (EAM) and viral (CVB3) myocarditis models, which recapitulate chronic inflammation and fibrosis that are commonly observed in patients with chronic myocarditis.

## Methods

### Human sample collection and bioinformatic analysis

The human heart samples were obtained from patients who underwent heart transplant surgery (**Table S1**). All harvested human hearts were cardio-protected using *in situ* cardiac paralysis. *In vivo* echocardiography was performed before tissue collection to allow real-time assessment of *in vivo* structure and function. The clinical investigation was carried out in accordance with the principles of the Declaration of Helsinki and was approved by the ethics committee review board of the Fuwai Hospital Shenzhen (Approval No.: 2022081-01). All participants provided written informed consent prior to inclusion in the study. Tissue specimens were frozen in liquid nitrogen and stored at -80 °C before use.

Total RNA was extracted from human cardiac tissue samples using the miRNeasy Kit (Qiagen) including DNase treatment. For RNA-sequencing analyses, library preparation was conducted using the Illumina truSeq stranded mRNA kit followed by the Nugen Ovation amplification kit. Resultant FASTQ files were assessed for quality control using the FastQC program. FASTQ files were aligned to the human reference genome (hGRC37/Hg19) using the STAR aligner 14. Duplicate reads were flagged using the MarkDuplicates program of Picard tools. Per gene read counts for Ensembl (v.75) gene annotations were computed using the R package with duplicate reads removed. Gene counts represented as counts per million (CPM) were first nominalized using the TMM method in the edgeR R package and genes with 25% of samples with a CPM < 1 were removed and deemed to have low expression. The data were transformed using the VOOM function of the limma R package 15. Differential gene expression was performed using a linear model using the limma package.

The data source of heat map use bulk RNA-seq of left ventricular tissues from 19 patients (4 with chronic myocarditis and 15 with dilated cardiomyopathy) and 15 healthy controls. Differential expression analysis was performed using the edgeR package in Bioconductor 3.19 (R 4.1.1), comparing each sample with the 15 healthy controls to obtain Log2 fold-change. The “fibroblast genes” were directly taken from the core feature gene set of human cardiac fibroblasts 4. The heatmap was plotted with z-score standardized Log2 fold-change using the R package ComplexHeatmap v2.10.0.

### Mouse models of chronic myocarditis

The Animal Ethics and Welfare Committee of Fuwai Hospital, Chinese Academy of Medical Sciences Shenzhen (Approval No.: 2022-032), approved all animal experiments involved in this study. Six-week-old BALB/c mice were used for autoimmune (EAM) and viral myocarditis models. For EAM, mice received subcutaneous α-MyHC peptide (250 μg) emulsified in CFA on days 0 and 7. Mice were divided into five groups: (1) Healthy controls (saline injections), (2) EAM only, (3) EAM + Captopril (10 mg/kg/day in drinking water from day 14), (4) EAM + Ctrl-T (non-targeting CAR-T cells on same schedule), and (5) EAM + FAP.CAR-T (5×10^6^ cells/mouse administered intravenously on days 14 and 28). All groups underwent terminal procedures at day 42, where cardiac tissues were harvested for fibrosis assessment, flow cytometry analysis of FAP^+^ fibroblasts, and systemic toxicity screening in major organs.

While CVB3 infection was established via intraperitoneal injection of 10^5^ TCID_50_ CVB3 on day 0. Mice were divided into three groups: (1) Healthy controls (saline injections), (2) CVB3 only, (3) CVB3 + Captopril (10 mg/kg/day in drinking water from day 7), (4) CVB3 + Ctrl-T (non-targeting CAR-T cells on same schedule), and (5) CVB3 + FAP.CAR-T (days 7 and 21). All groups underwent terminal procedures at day 35, where cardiac tissues were harvested for fibrosis assessment and immunohistochemical staining.

### CAR constructs and retroviral production

Murine and human FAP-targeted CARs were cloned into MSCV (mouse) or SFG (human) retroviral vectors, incorporating CD28/CD3ζ signaling domains. Retrovirus were produced in HEK293T cells using pCL-Eco (mouse) or PegPam/RDF114 (human) packaging systems.

### Generation and functional validation of murine CAR-T cells

Splenocytes were harvested from 6-8 weeks BALB/c mice and T cells were sorted by MojoSort Mouse CD3 T Cell Isolation Kit (Biolegend). Sorted T cells were then cultured in mouse T cell medium (RPMI 1640 + 10% FBS + 2mM Glutamax + 100U/mL Pen/Strep + 55mM b-mercaptoethanol + 1X NEAA + 1mM sodium pyruvate), stimulated with plate-bound anti-mouse CD3/CD28 antibodies (eBioscience) for two days. Activated mouse T lymphocytes were transduced with retroviral supernatant using retronectin-coated plates. 2 days post transduction (Day 4), T cells were collected and expanded in mouse T cell medium containing rhIL-7 (10 ng/ml) and rhIL-15 (5 ng/mL). The transduction efficiency of CAR was measured by surface detection of FLAG tag. All functional assays and *in vivo* experiments were conducted on Day 5 16.

Functional validation of murine CAR-T cells was conducted by co-culturing 2.5x10^5^ untransduced (Ctrl) or FAP.CAR-T cells with 5x10^4^ FAP^+^/FAP^-^ tumor cells (E:T = 5:1). T cell activation and cytokine production were measured 24hrs post co-culture and specific lysis of tumor cells were measured 3 days post co-culture. The specific lysis was calculated based on numerical counts of tumor cells cultured alone (Tumor only) or with Ctrl/FAP.CAR-T cells using this formula: (Tumor only - Ctrl/FAP.CAR-T cells)/Tumor only x 100%.

### Generation of human CAR-T cells

Human PBMCs were stimulated with plate-bound anti-CD3 and anti-CD28 antibodies for 2 days in human T cell medium (½ RPMI 1640 + ½ Click's Medium + 10% FBS + 2mM Glutamax + 100U/mL Pen/Strep) and transduced with retroviral supernatant using retronectin-coated plates. 3 days post transduction, cells were collected and positive transduction were confirmed by FLAG staining. Cells were then cultured in T human cell medium containing rhIL-7 (10 ng/ml) and rhIL-15 (5 ng/ml) for additional 7-10 days before functional assays 16. Functional validation of human FAP.CAR-T cells was conducted by co-culturing Ctrl or FAP.CAR-T cells with Daudi cells over-expressing human FAP protein. T cell activation were measured 24hrs post co-culture and numeration of tumor and T cells were performed 3 days post co-culture.

### Generation of human lymphoma cell line expressing murine or human FAP protein

Retrovirus encoding murine *Fap* or human *FAP* cDNA were produced using amphotropic packaging vector (RDF114) and transduced a human lymphoma cell line (Daudi) using spinoculation. Murine FAP (mFAP) expression was detected based on bicistronic-expressed DNGFR and the staining of surface mFAP by anti-mouse FAP monoclonal antibodies (clone 73.3, Millipore). DNGFR positive Daudi cells were fluorescence-activated cell sorting (FACS)-sorted to enrich for mFAP positive cells. For human FAP (hFAP), positively transduced Daudi cells were selected using 10 μg/mL blasticidin for 14 days and hFAP expression was confirmed by staining of anti-human FAP monoclonal antibodies (F11-24, Thermo), followed by Alexa-Fluor 647-conjugated goat-anti-mouse IgG.

### Flow cytometry

For cell-surface staining, cells were incubated with antibodies at room temperature for 15 min or at 4 °C for 30 min. The following antibodies used for the flow cytometry analysis were obtained from Biolegend: APC-conjugated anti-human CD20 (clone 2H7), BV421-conjugated anti-FLAG (clone L5), FITC-conjugated anti-mouse CD69 (clone H1.2F3), Alexa-Fluor 700-conjugated anti-mouse CD8 (clone 53-6.7), PE-Cy5-conjugated anti-mouse CD4 (clone Gk1.5), PE-conjugated anti-human NGFR (clone ME20.4), Alexa-Fluor 647-conjugated goat-anti-mouse IgG (clone Poly4053); PE-conjugated anti-human CD69 (clone FN50). The following antibodies were obtained from BD Bioscience: PE-conjugated anti-mouse CD3 (clone 500A2). The following antibodies were obtained from Thermo eBioscience: APC-conjugated anti-human CD3 (clone OKT3).

FACS data were collected on a Novocyte Quanteon (Agilent) flow cytometer using NovoExpress software, and the FACS data were analyzed with FlowJo software (v10.6.2, Tree Star).

### Echocardiography Performance and Analysis

Transthoracic echocardiography was performed using a Vinno 6 system (VINNO) with a 30-MHz transducer. Mice were anesthetized with 1.5% isoflurane in oxygen to maintain heart rates of 450-550 bpm. Data acquisition and analysis were performed by two investigators blinded to treatment groups. LV ejection fraction (EF) was calculated from M-mode images in parasternal long-axis views using Vinno Lab software, averaging three consecutive cardiac cycles.

### Flow cytometry

At the end of treatment in the mouse EAM model, fresh heart tissue was collected from each group of mice (n = 3). Fresh cardiac tissue samples were cut into small pieces in PBS at 4 ℃, followed by digestion in 250 U/ml type II collagenase (YEASEN, 40508ES60) at 37 °C water bath with mild shaking for 30 min. The single-cell suspension was filtered with a 40 μm cell strainer and collected by centrifugation at 300 × g for 5 min. The supernatant was discarded, and red blood cells were removed by RBC lysis (C3702, Beyotime). All prepared cells were suspended in PBS with 10% FBS. Mouse FAP Alexa Fluor® 647-conjugated Antibody (R&D Systems, FAB9727R, 0.25 μg/10^6^ cells) was added and incubated for 30 min in the dark. Finally, labeled cells were washed twice and resuspended in PBS with 10% FBS. Flow cytometry analysis was performed on a DxFLEX Flow cytometer (Beckman Coulter). We obtained data by using CytExpert software and analyzed data with FlowJo (10.0).

### Long-term safety assessment

For the long-term safety evaluation, ten EAM-injured mice treated with FAP.CAR-T (5×10^6^ cells per mouse) twice. The mice were maintained for 5 months, after which blood was collected for serum biochemistry analysis. Major organs (liver, spleen, lungs, kidneys, testicles, intestines, skeletal muscles, and pancreas) were harvested for histopathological assessment by standard H&E staining.

### Human cardiac organoids construction and myocardial fibrosis modeling

Cardiac cells were produced using recently developed protocols 17, 18. hiPSCs were seeded at 2 × 10^4^ cells/cm^2^ in Matrigel-coated flasks and were dissociated to single cells until 70%-80% cell confluency was reached. The cell suspension was added into an ultra-low attachment 96-well plate (Corning) with mTeSR Plus (Stem Cell Technologies) to form embryoid bodies (EBs). The next day diameter of spontaneously formed EBs was measured to estimate time of differentiation start. If critical diameter (300-500 μm) was reached, they were then differentiated into cardiac mesoderm using RPMI 1640 with B27 medium (without Insulin) supplemented with 5 ng/ml BMP-4 (R&D Systems), 9 ng/ml Activin A (R&D Systems) and 7 μM CHIR99021 (STEMCELL Technologies) with daily medium exchange for 3 days. Subsequently, cardiac organoids were transferred into an ultra-low attachment 6-well plate and the complete medium was changed to RPMI 1640 with B27 medium (with Insulin) supplemented with 5 μM IWP-4 (STEMCELL Technologies) with medium exchange every 2-3 days, which was placed on an orbital shaker with the speed of 100 rpm. The size of EBs would be larger than 800 μm. After 7 days, cells were cultured in RPMI 1640 with B27 medium (with Insulin) and 50% medium was refreshed every 3 days.

After matured, cardiac organoids were treated with 50 ng/ml rhIL-17 (Peprotech) for 7 days, while setting up the control group without IL-17. Subsequently, each cardiac organoid was treated with 4 × 10^4^ FAP.CAR-T cells for 5 days, while the group without FAP.CAR-T cells was also retained.

### Histological evaluation and staining

Formalin-fixed tissues were sectioned for H&E, Masson's trichrome, and immunofluorescence (FAP, CD68, MPO, LY6C, CD3, CD4, and CD8). Immunohistochemical used antibodies listed in **Table S3**.

### Statistical analysis

Statistical analyses were performed using GraphPad Prism 9.5.0. Non-parametric tests (Mann-Whitney U for two groups; Kruskal-Wallis with Dunn's correction for ≥ 3 groups) were prioritized due to small sample sizes. Parametric tests (ANOVA) were only applied to data passing normality checks (Shapiro-Wilk p > 0.05). All values are presented as the mean ± SD; n refers to the sample size. A value of p < 0.05 was considered statistically significant.

## Results

### Screening and validation of FAP as specific target of pro-fibrotic cells during myocarditis

To identify fibrosis-associated genes in myocarditis, we performed bulk RNA sequencing on left ventricular tissues from 19 patients (4 chronic myocarditis, 15 dilated cardiomyopathy (DCM)) and 15 healthy donors (**Table S1**). While clinical characteristics (age, gender, arrhythmia) were comparable between disease groups, DCM patients showed higher NT-proBNP levels (3,823.3 ± 1,734.9 vs. 1,622.2 ± 264.0 fmol/mL; P < 0.001), whereas myocarditis patients exhibited greater interventricular septal thickening and reduced LVEF/LVEDD.

**Figure 1:**
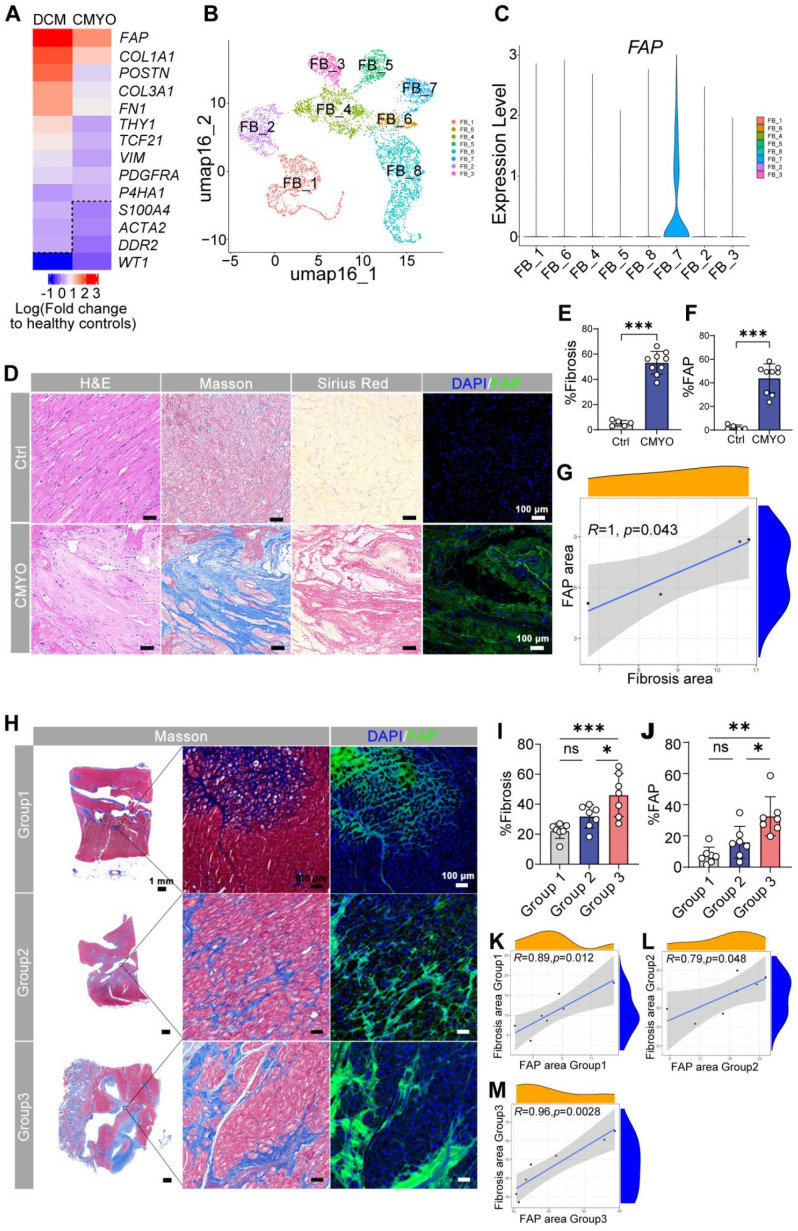
** Human cardiac fibroblast targets in disease.** (**A**) Heat map of cardiac fibroblast gene-expression changes (fold change) in patients with DCM and CMYO compared with non-failing hearts. n = 15 DCM hearts, 4 CMYO hearts. (**B**) Umap plot of cardiac fibroblasts clusters of heart failure and normal conditions data from previous study (GSE145154). (**C**) Violin plots of FAP expression in fibroblasts clusters. (**D**) H&E, Masson's trichrome stain of fibrosis (blue), Sirius Red stain of fibrosis (red), and immunohistochemistry of FAP (green) expression and DAPI (blue) stained in left ventricular free-wall sections from individual human samples of non-failing hearts and MYO (n_Ctrl_ = 5, n_CMYO_ = 9). Representative images of two independent experiments, showing similar results. (**E-F**) Quantification of ventricular fibrosis and FAP expression. (**G**) The correlation between fibrosis percentage and the FAP expression. (**H**) Masson trichrome staining (blue) for fibrosis and immunohistochemistry for FAP (green) expression and DAPI (blue) staining in left ventricular free wall sections from 21 human CMYO samples of varying CMYO duration (Group 1: <1 year; Group 2: 1~6 year; Group 3: 6~12 year). (**I-J**) Quantification of ventricular fibrosis and FAP expression in (H). (**K-M**) The correlation between fibrosis percentage and the FAP expression in each group of (H). Data are represented as mean ± SD. Statistical significance was calculated by two-tailed unpaired Student's t-test in (E, F), and by ordinary one-way ANOVA in (I, J), ns = not significance, *P < 0.05, **P < 0.01, ***P < 0.001.

Fibroblast-specific genes were upregulated in both myocarditis and DCM versus controls (**Figure 1A**), with *FAP* showing the largest fold change. scRNA-seq data from our prior heart failure study (GEO: GSE145154) 19 confirmed *FAP* preferentially enriched in activated myofibroblasts (FB_7 cluster; **Figure 1B-C**), which is consistent with what was reported among the studies 20, 21. Immunohistochemistry validated FAP protein enrichment in diseased hearts (**Figure 1D-F, Figure S1**), strongly correlating with fibrosis severity (r = 0.82; **Figure 1G**).

Expanding to 21 myocarditis patients stratified by disease duration (<1, 1-6, >6 years; **Table S2**), we observed progressive fibrosis (**Figure 1H-I**) and FAP upregulation (**Figure 1J**), peaking in chronic cases (>6 years). Fibrosis extent directly correlated with FAP expression across all groups (**Figure 1K-M**), nominating FAP as a therapeutic target for myofibroblast-driven pathology. Additionally, histological quantitative analysis of 30 additional myocarditis cases confirmed that FAP^+^ areas were significantly correlated with fibrosis severity (r^2^ = 0.904, P < 0.0001, **Figure S2**).

### Murine FAP-specific CAR T cells

**Figure 2:**
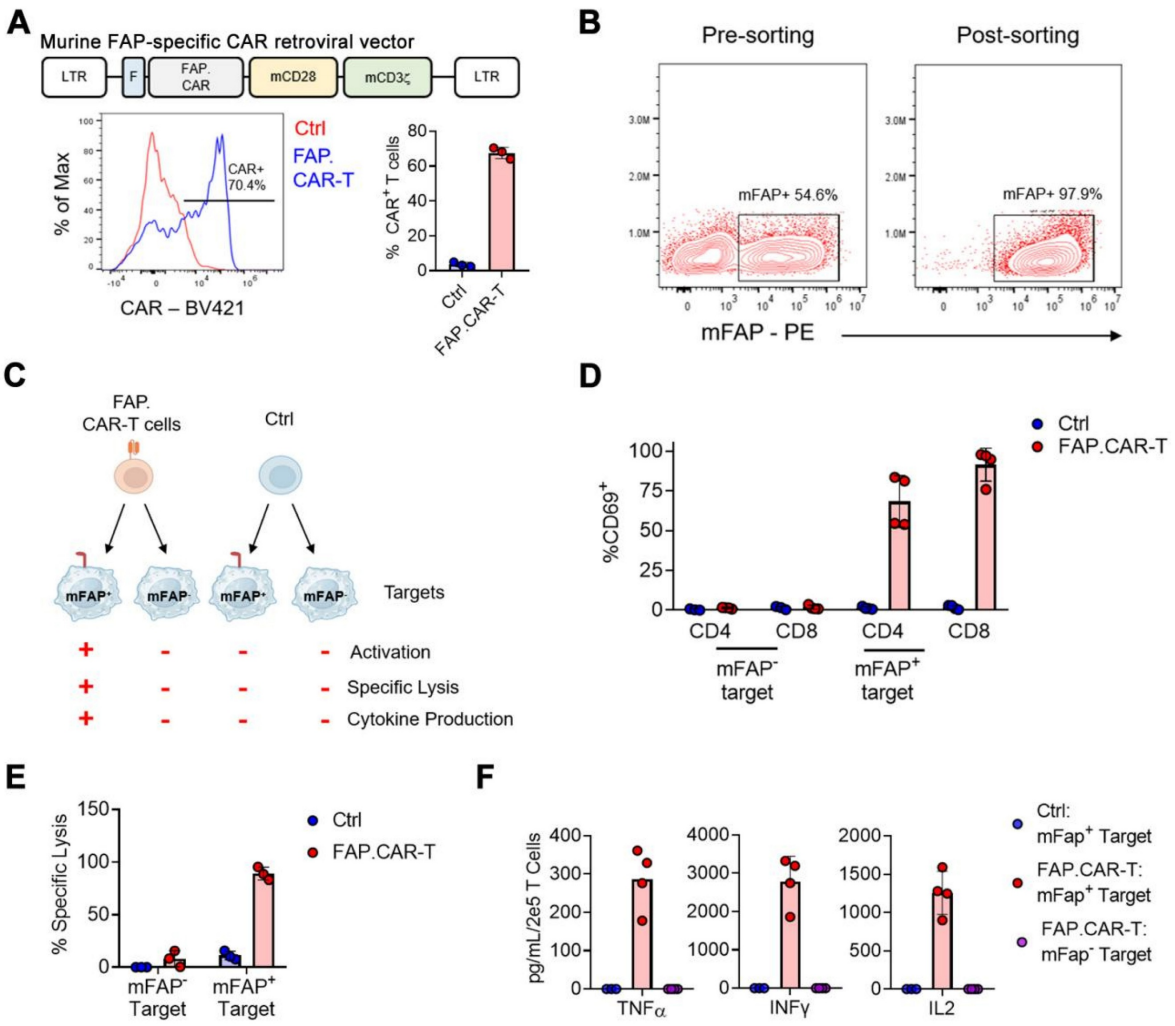
** Construction and validation of murine FAP-targeted CAR T cells.** (**A**) Retroviral construct encoding a CAR molecule specific against FAP, representative and summary of CAR expression in murine T cells. n = 3; (**B**) Over-expression of murine *Fap* gene and FACS sorting of mFAP positive target cells; (**C**) Schematics of functional experiments validating the specificity of murine FAP.CAR-T cells; (**D**) Expression of CD69 activation markers on CD4^+^ and CD8^+^ T cells upon co-culture with mFAP positive or negative targets. n = 3 for Ctrl and n = 4 for FAP.CAR-T cells; (**E**) Percentage of lysis of mFAP positive or negative targets upon co-culture with Ctrl or FAP.CAR-T cells. n = 3 for Ctrl and n-4 for FAP.CAR-T cells; (**F**) Production of cytokines by Ctrl or FAP.CAR-T cells upon co-culture with mFAP positive or negative targets. n = 4.

We hypothesize that targeted elimination of FAP positive cells could ameliorate the pathologic fibrosis that accumulates in chronic myocarditis. Therefore, we constructed a CAR molecule targeting mouse FAP (referred to as FAP.CAR), as shown in the schematic diagram in **Figure 2A-top**. Flow cytometry results showed that the CAR molecule was efficiently expressed (**Figure 2A-bottom**). To validate functions of expressed CAR molecules, we engineered a human lymphoma cell line (Daudi) that ectopically express mFAP and sorted surface mFAP positive cells by FACS (**Figure 2B**). Using this mFAP positive cell line as well as wild type cell line that lacks mFAP expression, we tested the specificity and functional targeting of FAP.CAR-T cells (**Figure 2C**) with untransduced T cells as control (Ctrl). We found FAP.CAR-T cells were specifically activated by mFAP positive target cells based on CD69 expression, while Ctrl T cells remained unstimulated (**Figure 2D**). Moreover, the activation of FAP.CAR-T cells led to an effective eradication of mFAP positive target cells but not mFAP negative control (**Figure 2E**). Consistently, FAP.CAR-T cells also robustly produced cytokines (TNF-a, IFN-g and IL-2) upon co-culture with mFAP positive target cells (**Figure 2F**). Overall, these experiments confirmed that our FAP.CAR-T cells could effectively target and lyse cells expressing murine FAP protein.

### FAP.CAR-T cells reverse fibrosis and restore cardiac function in myocarditis models

**Figure 3:**
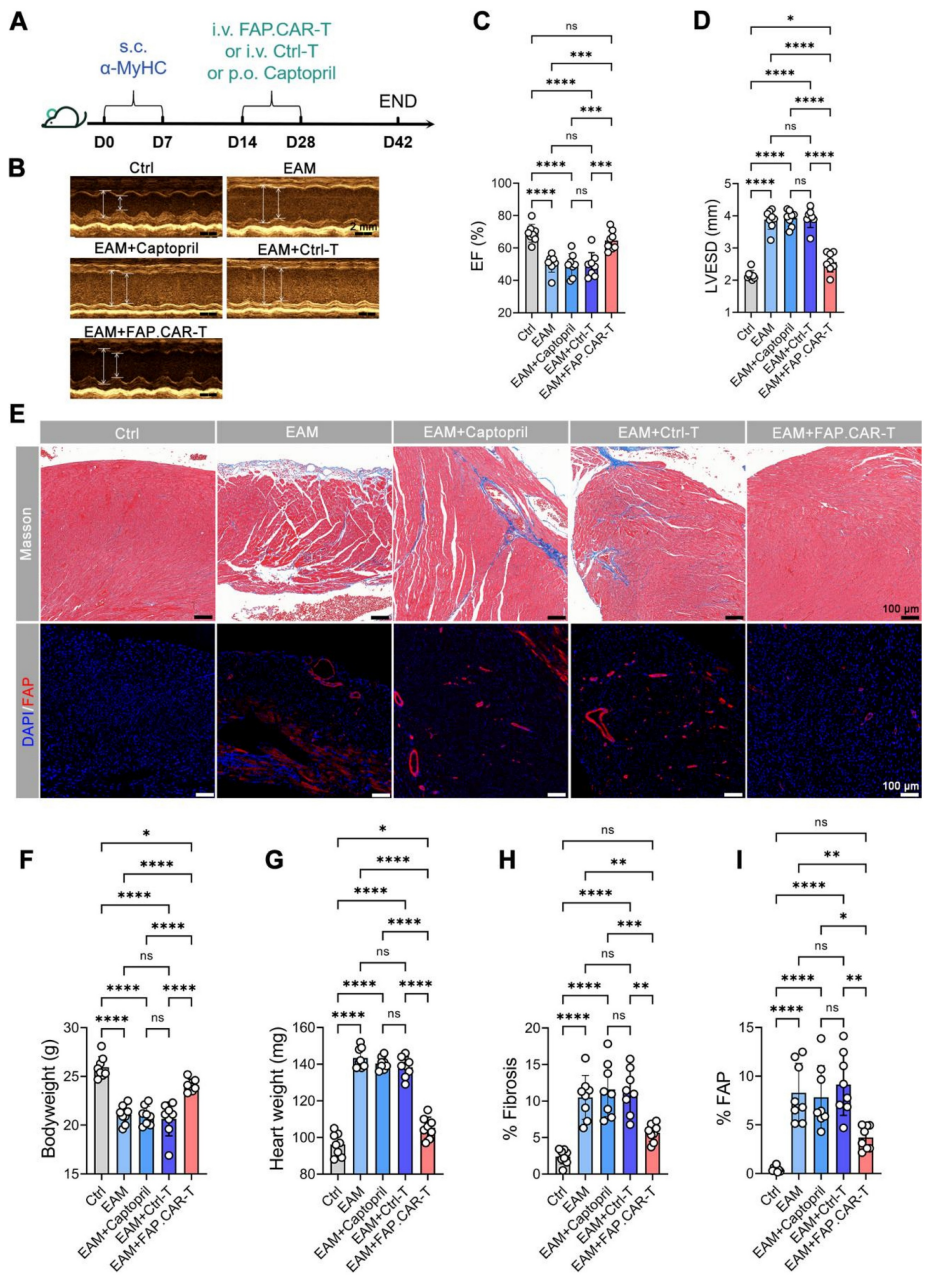
** FAP.CAR-T cells target mouse cardiac fibrosis in EAM model.** (**A**) Schematic of experiments for FAP.CAR-T cells targeting of cardiac fibroblasts in EAM model. (**B**) M mode echocardiography of mice in different treatment groups. Scale bars, 2 mm. (**C, D**) Statistics of ejection fraction (EF) and LVESD of each group. (**E**) Coronary sections of mouse hearts were stained for Masson's trichrome staining for fibrosis (blue), and immunofluorescence staining for DAPI (Blue) and FAP (red) in different treatment groups: EAM, EAM+Captopril, EAM+Ctrl-T, EAM+FAP.CAR-T. Healthy mice were used as controls. Scale bars, 100 μm. (**F, G**) Body weight and heart weight of mice in different treatment groups. (**H, I**) Quantification of ventricular fibrosis and FAP expression. n = 8 in biologically independent mice per group. Data are represented as mean ± SD. Statistical significance was calculated by ordinary one-way ANOVA, ns = not significance, *P < 0.05, **P < 0.01, ***P < 0.001, and ****P < 0.0001.

**Figure 4:**
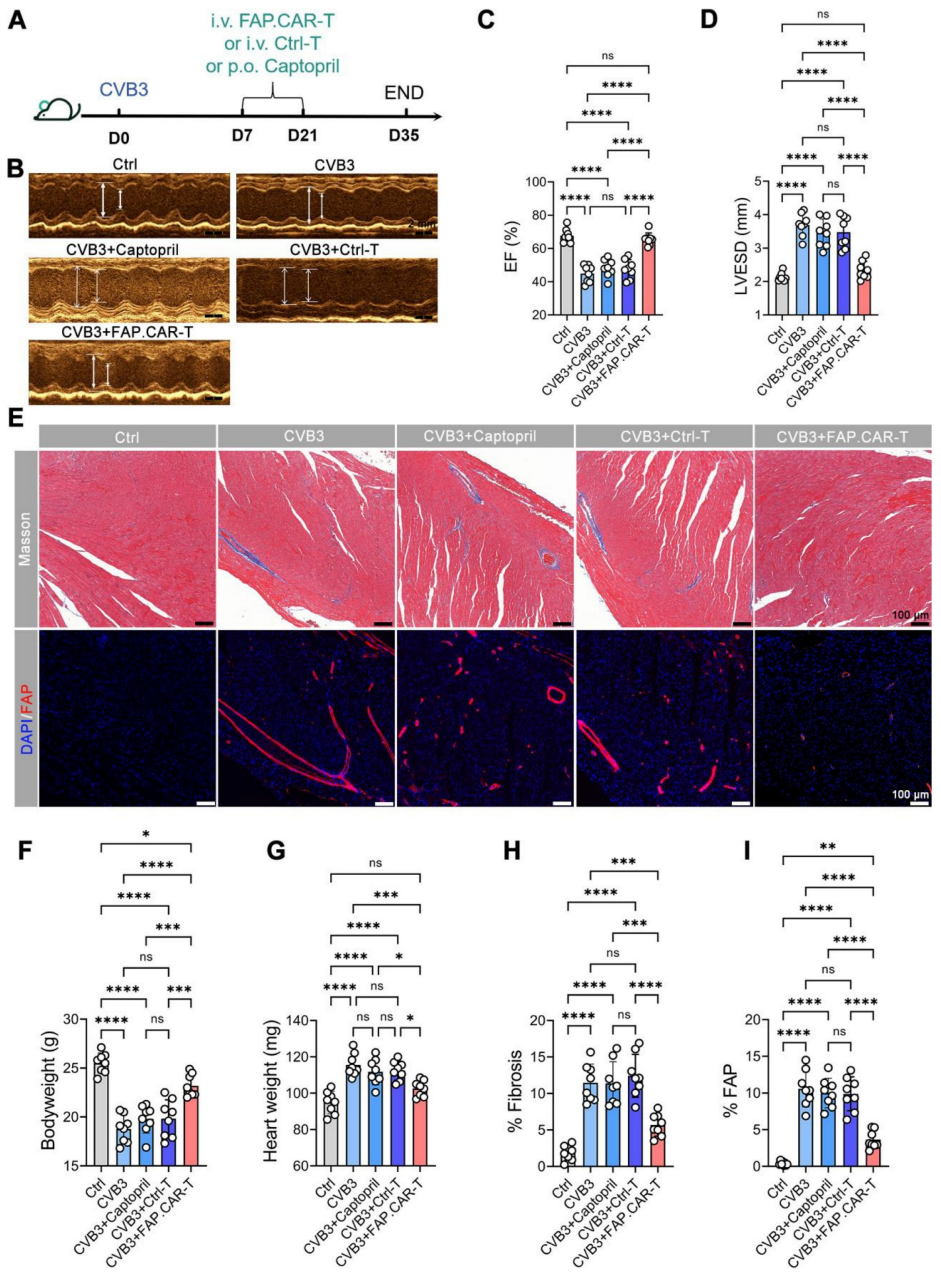
** FAP.CAR-T cells target mouse cardiac fibrosis in CVB3-induced mouse myocarditis model.** (**A**) Schematic of experiments for FAP.CAR-T cells targeting of cardiac fibroblasts in CVB3-induced mouse myocarditis model. (**B**) M mode echocardiography of different group of mice. Scale bars, 2 mm. (**C, D**) Statistics of ejection fraction (EF) and LVESD of each group. (**E**) Coronary sections of mouse hearts were stained for Masson's trichrome staining for fibrosis (blue), and immunofluorescence staining for DAPI (Blue) and FAP (red) in different treatment groups: CVB3, CVB3+Captopril, CVB3+Ctrl-T, CVB3+FAP.CAR-T. Healthy mice were used as controls. Scale bars, 100 μm. (**F, G**) Body weight and heart weight of mice in different treatment groups. (**H-I**) Quantification of ventricular fibrosis and FAP expression. n = 8 in biologically independent mice per group. Data are represented as mean ± SD. Statistical significance was calculated by ordinary one-way ANOVA, ns = not significance, *P < 0.05, **P < 0.01, ***P < 0.001, and ****P < 0.0001.

Myocarditis typically arises from two main causes: autoimmune disorders and viral infections 22. Hence, we developed two distinct models for chronic myocarditis: the EAM model and coxsackievirus B3 infected model and tested the therapeutic efficacy of FAP.CAR-T cells (**Figure 3A, 4A**). FAP expression at the treatment time point (day 14 of EAM) was confirmed by immunohistochemical staining of cardiac tissue (**Figure S3**). This confirmed the presence of early activated of FAP^+^ fibroblasts prior to CAR-T therapy. Notably, FAP.CAR-T cell-mediated clearance of fibrotic cells significantly restored cardiac function and reversed pathological remodeling. Compared to healthy controls, the EAM model exhibited severely impaired systolic function, as evidenced by a reduction in ejection fraction (EF, 38 ± 3% vs. 69 ± 2% in controls) and elevated left ventricular end-systolic dimension (LVESD; **Figure 3D**). Treatment with FAP.CAR-T cells effectively reversed these deficits, restoring EF to near-normal levels (65-72%, comparable to healthy controls; **Figure 3B-C, 4B-C**) and reducing LVESD by 28.3 ± 4.1% (P < 0.001; **Figure 3D, 4D**). Furthermore, EAM-induced weight loss was substantially recovered after therapy (**Figure 3F, 4F**), accompanied by 21.7% (EAM model) and 12.2% (CVB3 model) decrease in heart weight (**Figure 3G, 4G**). Histological analysis confirmed these functional improvements: fibrosis area was reduced by approximately 65% (EAM) and 55% (CVB3) (both P < 0.001; **Figure 3H, 4H**), while FAP^+^ fibroblasts were selectively eradicated as shown by immunofluorescence and flow cytometry (**Figure 3E&I, Figure S5, Figure 4E&I**), confirming the *in viv*o functionality of the infused CAR-T cells. When EAM and CVB3-induced mice were treated with untransduced T cells (Ctrl) or with standard of care ACE inhibitor Captopril, no significant anti-fibrotic inhibition was observed under the present conditions (**Figure 3B-I, 4B-I**). Concomitant with fibrosis reduction, FAP.CAR-T cell treatment significantly attenuated myocardial infiltration of pro-inflammatory cells, including CD68^+^ macrophages, LY6C^+^ monocytes, and MPO^+^ neutrophils, compared to disease controls (**Figure S4A-C; Figure S6A-C**). Notably, we observed a marked increase in T cells infiltration in the myocardium of FAP.CAR-T-treated mice (**Figure S4D-E, S6D-E**).

### *In vivo* safety evaluation of FAP.CAR-T cells

**Figure 5:**
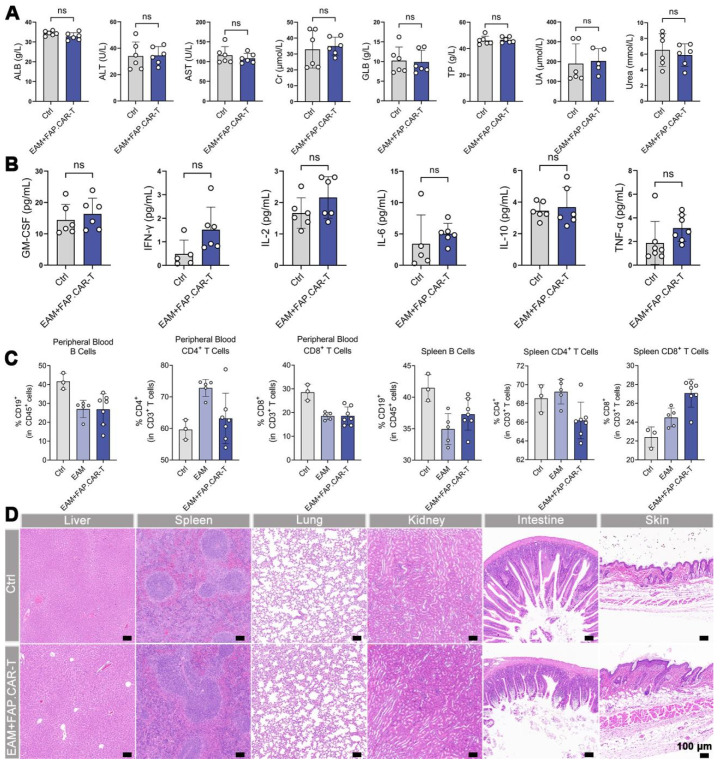
***In vivo* biosafety evaluation.** (**A**) Blood biochemistry indexes of mice after treatments (Ctrl: Healthy mice i.v. injection of saline, n = 6; EAM + FAP.CAR-T: EAM-injured mice treated with FAP.CAR-T, n = 6). (**B**) Quantitative analysis of TNF-α, IFN-γ, IL-2, GM-CSF, IL-6, and IL-10 for the mice in different groups by ELISA (Ctrl: Healthy mice i.v. injection of saline, n = 6; EAM + FAP.CAR-T: EAM-injured mice treated with FAP.CAR-T, n = 6). (**C**) FACS analysis of heart tissue, peripheral blood, and spleen of FAP.CAR-T cells treated mice. (Ctrl: Healthy mice i.v. injection of saline, n = 3; EAM: Disease controls with EAM injury, no treatment, n = 5; EAM + FAP.CAR-T: EAM-injured mice treated with FAP.CAR-T, n = 7). (**D**) H&E staining of various tissue sections from mice with different treated. (Ctrl: Healthy mice i.v. injection of saline, n = 3; EAM + FAP.CAR-T: EAM-injured mice treated with FAP.CAR-T, n = 7). Representative images of two independent experiments, showing similar results. Scale bars, 100 μm. Data are represented as mean ± SD. Statistical significance was calculated by two-tailed unpaired Student's t-test in (A, B), and by ordinary one-way ANOVA in (C), ns = not significance.

In EAM-injured mice treated with FAP.CAR-T cells, no significant alterations were observed in blood biochemistry, cytokine levels (TNF-α, IL-6; **Figure 5A-B**), or immune cell proportions in peripheral blood and spleen (**Figure 5C**) at the study endpoint. Histopathological assessment of non-cardiac organs from these mice also revealed no lesions (**Figure 5D**). Furthermore, a long-term safety evaluation in EAM-injured mice treated with FAP.CAR-T cells did not induce hepatorenal toxicity or tissue damage after a 5-month observation period (**Figure S7A,B**). These collective findings indicate minimal off-target toxicity of the FAP.CAR-T therapy.

### Human FAP.CAR-T cells attenuate fibrosis in cardiac organoids

**Figure 6:**
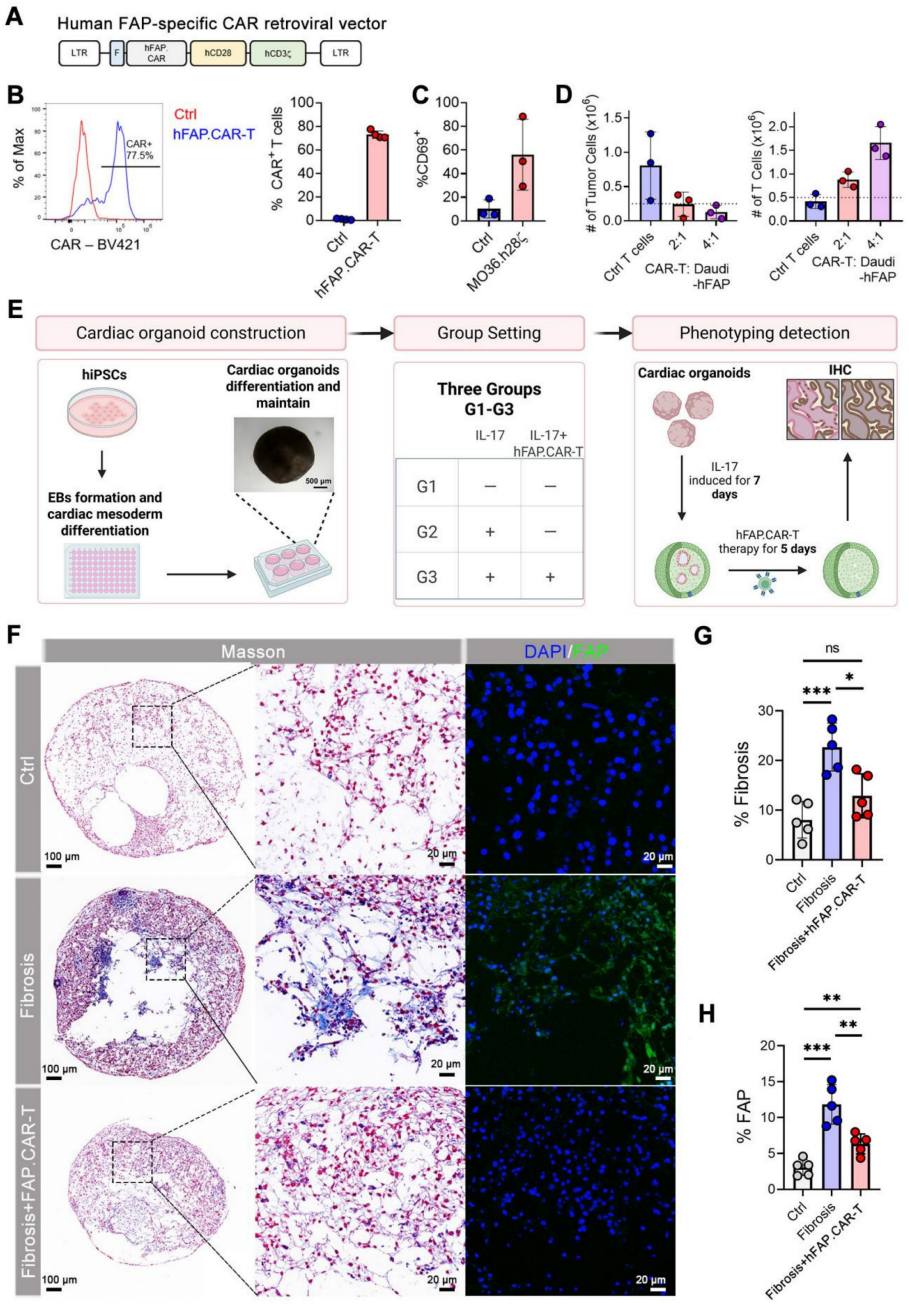
** Construction and validation of human FAP.CAR-T (hFAP.CAR-T) cells and *in vitro* evaluation in hCOs model.** (A) Retroviral construct encoding a CAR molecule specific against FAP. (B) Representative and summary of CAR expression in human T cells (n = 4). (C) Expression of CD69 activation markers on CAR T cells upon co-culture with FAP-transduced Daudi cells (N = 3). (D) Number of Tumor Cells and T cells upon FAP^+^ Daudi cells co-culture with Ctrl or hFAP.CAR-T cells (n = 3). (E) Schematic diagram of the *in vitro* evaluation process of human cardiac organoids. (F) Immunohistochemical staining of organoids. (G, H) Fibrosis and FAP expression statistics (n = 5). Data are represented as mean ± SD. Statistical significance was calculated by ordinary one-way ANOVA, ns = not significance, *P < 0.05, **P < 0.01, ***P < 0.001.

To facilitate the clinical translation of FAP.CAR-T cells in treatment of myocarditis, we constructed a CAR molecule targeting human FAP based on MO36 mAb clone by fusing its scFv fragment with human CD28 and CD3z endodomain (referred as hFAP.CAR, **Figure 6A**). hFAP.CAR could be efficiently expressed in primary human T cells (**Figure 6B**). To test the killing efficacy of hFAP.CAR, we transduced Daudi with human FAP cDNA and co-culture it with control (Ctrl) or hFAP.CAR-T cells. hFAP.CAR-T cells up-regulated T cell activation marker CD69 upon engagement with FAP positive Daudi cells (**Figure 6C**). In addition, hFAP.CAR-T cells could efficiently eradicate Daudi-FAP cells after 72 h co-culture (**Figure 6D**).

We investigated the effectiveness of FAP.CAR-T cells therapy in reducing fibrosis in human cardiac organoid (hCO) models. We first induced differentiation and cultured adult hCOs using human pluripotent stem cells (hPSCs). Immunofluorescence staining of hCOs tissue revealed that the organoids are enriched with cardiomyocytes (**Figure S8**), form a chamber-like structure (**Figure 6F, Figure S9**), and exhibit rhythmic contractility (**Video 1**). Once the organoids are stably generated, we added rhIL-17A, an inflammatory cytokine previously been implicated in autoimmune myocarditis and major driver of tissue reconstruction during myocarditis 23-25. Consistent with observations in patients and mouse models, rhIL-17A induced FAP expression and strong fibrotic phenotype in hCOs (**Figure 6F-H, Figure S10**), confirming the central role of IL-17A in inducing fibrosis in inflammatory myocarditis. hFAP.CAR-T cells effectively eradicated FAP positive cells and cleared the fibrotic phenotype. Fibrosis was reduced by 55% in the hFAP.CAR-Ts group after treatment, compared to the Fibrosis group. In summary, these data highlight the application of hCOs as a pre-clinical model to study inflammatory induced myocarditis and cardiac fibrosis and the impressive anti-fibrotic activity of our hFAP.CAR-T cells.

## Discussion

Our study establishes FAP.CAR-T cells as a potent strategy to reverse inflammation-driven cardiac fibrosis, addressing a critical unmet need in chronic myocarditis. By targeting FAP^+^ myofibroblasts in both autoimmune and viral models, we achieved >60% fibrosis reduction and near-complete functional recovery. Notably, this is the first demonstration of CAR-T therapy in myocarditis. The anti-inflammatory effects of FAP.CAR-T cells were unexpected. We speculate that clearing fibrotic niches may disrupt pathological crosstalk between myofibroblasts and immune cells. For instance, FAP^+^ cells secrete pro-fibrotic mediators (e.g., TGF-β, periostin) that recruit macrophages 26, and their elimination could indirectly dampen inflammation. While our data do not prove direct CAR-T-mediated immunomodulation, they align with recent work by Alexanian et al. demonstrating that fibroblast depletion alters immune cell dynamics through remodeling of the fibrotic niche 27. This reduction in inflammation is likely an indirect consequence of targeting pathogenic myofibroblasts. As established in recent research 28, chronic fibrosis and inflammation are interdependent: myofibroblasts actively secrete pro-inflammatory factors that recruit immune cells (e.g., macrophages, neutrophils). By efficiently clearing FAP^+^ myofibroblasts, CAR-T cells disrupt this vicious cycle-thereby reducing secondary immune cell infiltration without direct anti-inflammatory intervention. These immune changes strongly correlated with attenuated fibrosis (r = 0.86-0.89, **Figure 3E, 4E**), improved cardiac function (EF: 65-72%, **Figure 3B-C, 4B-C**), and no systemic toxicity (stable cytokines, **Figure 5B**). Future studies should map spatial fibroblast-immune networks in resolved fibrosis.

It is worth noting that we observed recovery of weight loss in mice following CAR-T therapy. We hypothesize that FAP.CAR-T reverses weight loss in EAM mice through a sequential recovery cascade: improved cardiac function (restored EF) enhances systemic perfusion, normalizing gastrointestinal activity and nutrient absorption; subsequent dietary recovery, combined with potential gut microbiota restoration, breaks the “inflammation-fibrosis-metabolic dysfunction” cycle.

Safety is paramount for translating CAR-Ts into non-cancer diseases. While Tran et al. reported FAP.CAR-T-induced cachexia via on-target/off-tumor toxicity 29, our construct (clone 73.3 scFv with CD28/CD3ζ) showed no adverse effects in mice or hCOs. This discrepancy may stem from differences in CAR design and deserve further study. Rigorous tissue-specific FAP screening in humans will be essential for clinical trials.

Our hCO model overcomes a key limitation of murine studies: species-specific fibroblast behaviors. Here, we use IL-17A to induce the fibrosis process of hCO. In autoimmune and viral myocarditis, IL-17A orchestrates fibroblast activation through epithelial-mesenchymal transition 30, 31, TGF-β synergy, and ECM cross-linking. This activation prompts fibroblasts to undergo a pathological shift into a FAP-positive, collagen-secreting phenotype, thereby sustaining fibrosis even after the initial inflammatory signals subside. Prior study revealed IL-17A receptor (IL17RA) overexpression in fibroblast clusters 32, positioning IL-17A as a keystone mediator connecting inflammation to fibrosis. IL-17-treated hCOs faithfully mimicked human myocardial fibrosis, and human FAP.CAR-T cells effectively cleared FAP^+^ cells without harming cardiomyocytes. This platform could accelerate preclinical testing of antifibrotic therapies. Although IL-17A alone reproduces key aspects of autoimmune-driven fibrosis, future organoid models will incorporate a multifactorial cytokine environment (e.g., TNF-α + TGF-β + IL-17A) to better mimic viral or mixed etiology myocarditis. Our hCO provides a platform to model cardiac fibrosis during inflammatory myocarditis, allowing us to assess the therapeutic potential of CAR-T cells in treating such condition. The FAP expression in non-cardiac tissues were documented in past studies 33 and “no or very low levels of FAP staining were detected except for organs with areas of remodeling tissue (proliferating endometrium, placenta).” Regarding the scalability of CAR-T production, since we are treating a relatively slow-progressing disease, the therapeutic dose of CAR-T cells is dramatically lower than that of CAR-T cells used to treat cancer. Furthermore, technologies that enable *in vivo* manufacture of CAR-T cells could also help expand the accessibility of the therapy.

Current cardiac fibrosis therapies primarily focus on targeting FAP, as demonstrated in key models like angiotensin II-induced fibrosis 4, 34 and myocardial infarction-induced fibrosis 35. While exploration of new targets is ongoing, novel candidates such as POSTN and SPP1 require deeper investigation 20; particularly, as these are not membrane proteins, their suitability for CAR-T cell therapy remains uncertain and warrants further study.

Recent advances demonstrate the efficacy of CAR-T therapy in autoimmune disorders like rheumatoid arthritis and systemic sclerosis 36, 37, where it targets pathogenic cells to regulate fibroblast activation and collagen deposition-directly alleviating fibrosis without broad immunosuppression. Extending these findings, our work specifically addresses inflammation-driven cardiac fibrosis, FAP.CAR-T uniquely eliminates activated myofibroblasts in autoimmune myocarditis, achieving fibrosis reduction and functional recovery (**Figure 3-4**). This validates translational potential of CAR-T therapy in autoimmune settings and advances the field through successful human organoid validation (**Figure 6**), highlighting a novel strategy for therapy-resistant fibrotic heart disease.

Although our study demonstrates potent short-term efficacy of FAP.CAR-T cells and preliminary long-term safety over 5 months in a murine model, the durability of anti-fibrotic benefits beyond this period remains to be established. Future studies employing longitudinal follow-up in immunocompetent large animal models will be essential to assess whether fibrosis recurrence occurs or whether compensatory pathways (e.g., TGF-β, POSTN, or other pro-fibrotic signals) emerge upon sustained FAP+ cell depletion. Future studies are needed to determine the mechanism of FAP.CAR-T-mediated clearance. Single-cell transcriptomics and high-resolution spatial profiling could clarify whether target cell elimination occurs via direct cytolysis, induction of apoptosis, functional silencing, or immune modulation. Such approaches could also reveal whether FAP.CAR-T cells alter the cardiac immune landscape durably, potentially breaking the cycle of inflammation and fibrosis beyond the acute treatment phase.

While FAP.CAR-T therapy demonstrated a favorable safety profile in our chronic myocarditis models, the physiological role of FAP^+^ fibroblasts warrants careful consideration. Emerging evidence suggests that transient myofibroblast activation facilitates tissue repair, whereas sustained activation drives pathological fibrosis 38. Our findings align with this paradigm, as selective depletion of persistently activated FAP^+^ myofibroblasts in chronic inflammatory settings reversed established fibrosis, restored ejection fraction to >65%, and normalized body weight without compromising cardiac structure. Recent studies further support the therapeutic value of targeting FAP^+^ cells, demonstrating that their ablation or inhibition can enhance repair in various cardiac injury models 39, 40. Beyond FAP, emerging fibroblast markers offer alternative targeting opportunities. CD248 (endosialin) has recently been identified as a key fibroblast subpopulation driving chronic fibrosis after myocardial infarction, where it participates in regulating the post-ischemic inflammatory microenvironment 41, 42. Notably, both CD248^+^ and FAP^+^ fibroblast ablation have demonstrated comparable efficacy in improving cardiac function in ischemic models 39. These parallel successes suggest a promising landscape for targeted fibroblast therapy, though the relative accuracy, effectiveness, and safety of FAP versus CD248 as CAR-T targets remain to be systematically compared in identical disease contexts. Future studies will specifically evaluate CD248-targeting strategies in chronic myocarditis models to determine whether combinatorial or context-dependent targeting approaches might optimize therapeutic outcomes across the spectrum of fibrotic cardiovascular diseases.

### Study limitations

There are several limitations warrant consideration in this study. First, the follow-up period was restricted to 8 weeks, which precludes an assessment of long-term outcomes such as the durability of fibrosis reduction, potential late-onset toxicities associated with persistent CAR-T cell activity, or the risk of fibrotic recurrence in treated tissues. These factors are critical for evaluating clinical viability, particularly given the chronic nature of myocarditis-driven heart disease. The temporal scope of our therapeutic efficacy studies was limited to 8 weeks post-treatment. Although we have incorporated a separate 5-month safety assessment that showed no chronic toxicity, this still precludes a definitive evaluation of long-term outcomes such as sustained fibrosis regression or fibrotic recurrence. Additionally, while we observed a significant reduction in inflammatory infiltrates post-treatment, the precise molecular mechanisms through which FAP.CAR-T cells exert these effects-e.g., via direct cytolytic activity, bystander immune modulation, or alteration of the fibroblast-immune crosstalk-remain incompletely elucidated. Future work incorporating longer-term observations (6-12 months), large animal models, and spatial multi-omics technologies will be critical to dissect these mechanisms and identify any compensatory pathways that may limit long-term efficacy. The preclinical validation was confined to murine models and human cardiac organoids, omitting large animal studies in species such as pigs or non-human primates. Besides, while a reduction in inflammatory infiltration was observed post-treatment, the exact mechanisms (such as direct interactions between CAR-T immune cells and secondary effects on fibrosis resolution) and the precise molecular regulators governing macrophage/neutrophil dynamics require further validation. While EAM and CVB3 models capture core autoimmune/viral etiologies, human myocarditis exhibits additional heterogeneity (e.g., checkpoint inhibitor-associated forms). Broad applicability of FAP.CAR-T therapy across subtypes warrants further investigation.

## Conclusion

In conclusion, FAP.CAR-T therapy eliminates fibrosis-driving myofibroblasts in chronic myocarditis, reversing cardiac dysfunction across murine and human models. By targeting FAP^+^ cells within inflammatory microenvironments, we achieved robust fibrosis resolution (>60%) and functional recovery without systemic toxicity. The success of human FAP.CAR-T cells in cytokine-treated cardiac organoids underscores their translational potential. Our study nominates FAP.CAR-T cells as a first-in-class immunotherapy for myocarditis patients progressing to fibrosis, particularly those unresponsive to conventional therapies. Future work should optimize CAR-T persistence and validate safety in non-human primates, paving the way for early-phase clinical trials.

## Supplementary Material

Supplementary figures and tables.

Supplementary HDO video.

## Abbreviations

CMYO: chronic myocarditis
EAM: experimental autoimmune myocarditis
scRNA-seq: single-cell RNA sequencing
FAP: Fibroblast activation protein
FAP.CAR-T cells: FAP- targeted CAR T cells
CVB3: coxsackievirus B3
