# Supplementary material for: Engineered T cell therapy for the treatment of cardiac fibrosis during chronic phase of myocarditis
Source: Theranostics. 2026 Jan 1;16(4):2037–51. doi: 10.7150/thno.116749 (PMC12680599; doi:10.7150/thno.116749)
Supplement: Supplementary file 1 — Supplementary figures and tables. [file thnov16p2037s1.pdf]

Supplemental Information

Engineered T cell therapy for the treatment of cardiac fibrosis during chronic phase of myocarditis

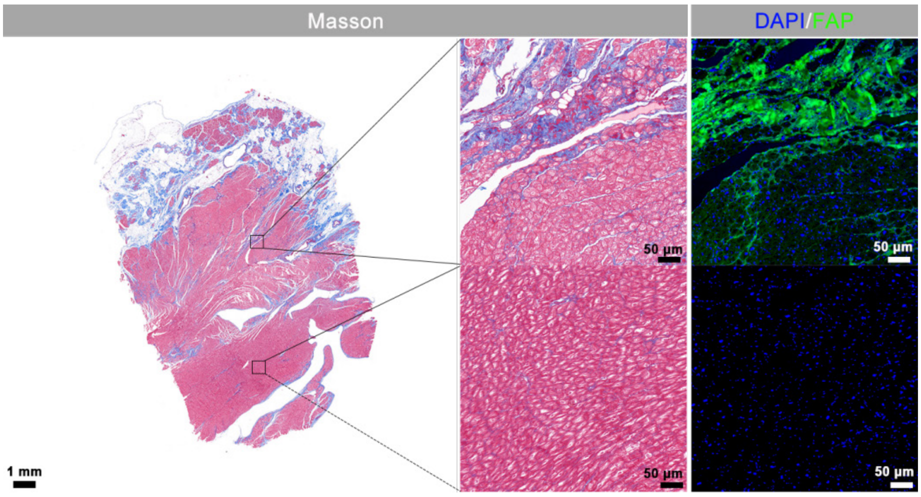

Figure S1. Representative images of Masson staining and immunofluorescence staining of myocardial samples from different regions of the left ventricle.

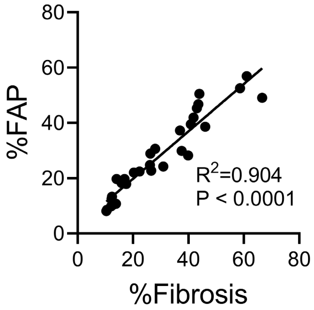

Figure S2. Statistical correlation between myocardial fibrosis and FAP expression in human myocarditis samples (n=30).

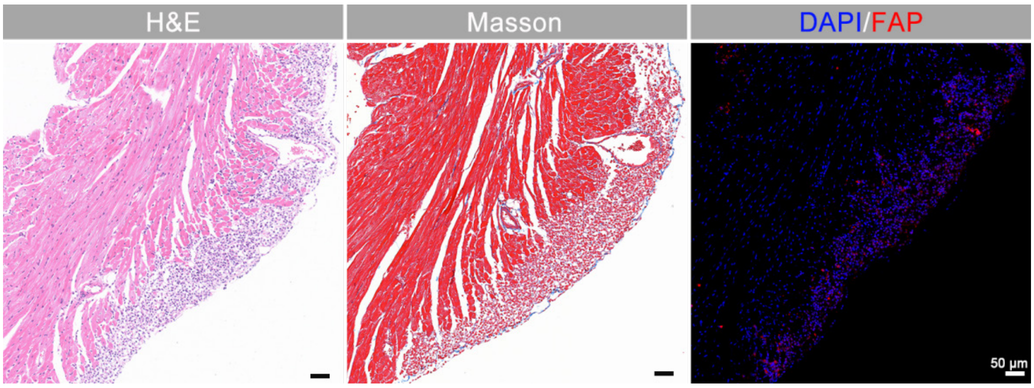

Figure S3. Representative immunohistochemical staining images from day 14 of the mouse EAM model.

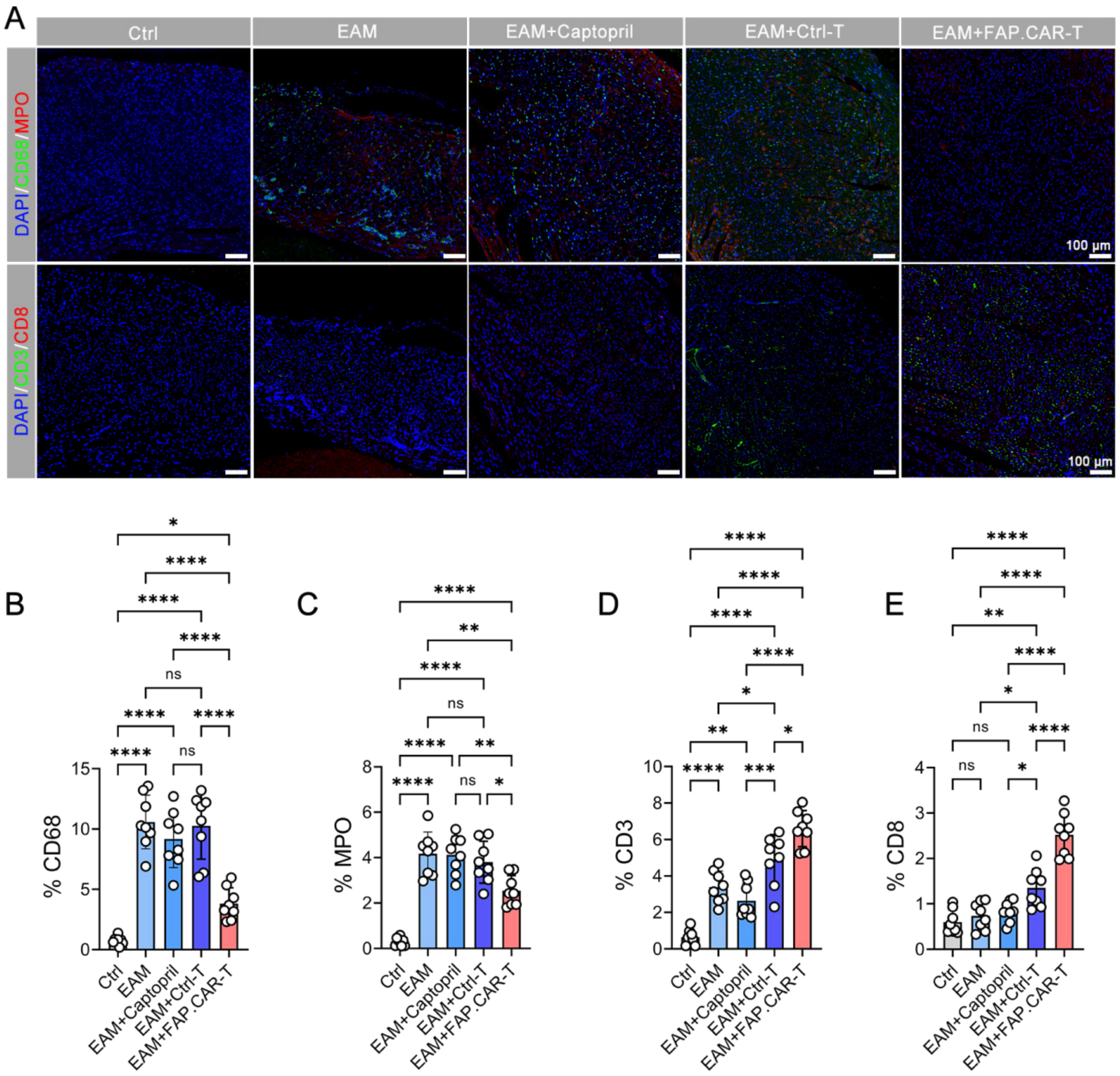

Figure S4. (A) Immunofluorescence staining images and (B-E) statistics of $CD68^+$, MPO^+ cells and $CD3^+$, and $CD8^+$ T cells in heart slices after different treatments in EAM model (n = 8 in biologically independent mice per group). Scale bars, 100 μm. Data are represented as mean ± SD. Statistical significance was calculated by ordinary one-way ANOVA, ns = not significance, *P < 0.05, **P < 0.01, ***P < 0.001, and ****P < 0.0001.

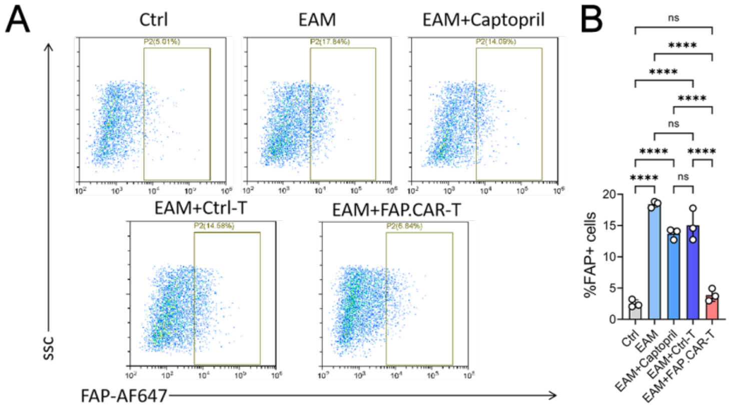

Figure S5. (A) Flow cytometry of fibroblasts isolated from hearts of BALB/C WT and EAM mice with different treatment. Typical images show FAP^+ fibroblasts ratio. (B) Quantifying the FAP^+ fibroblast ratio (n = 3).

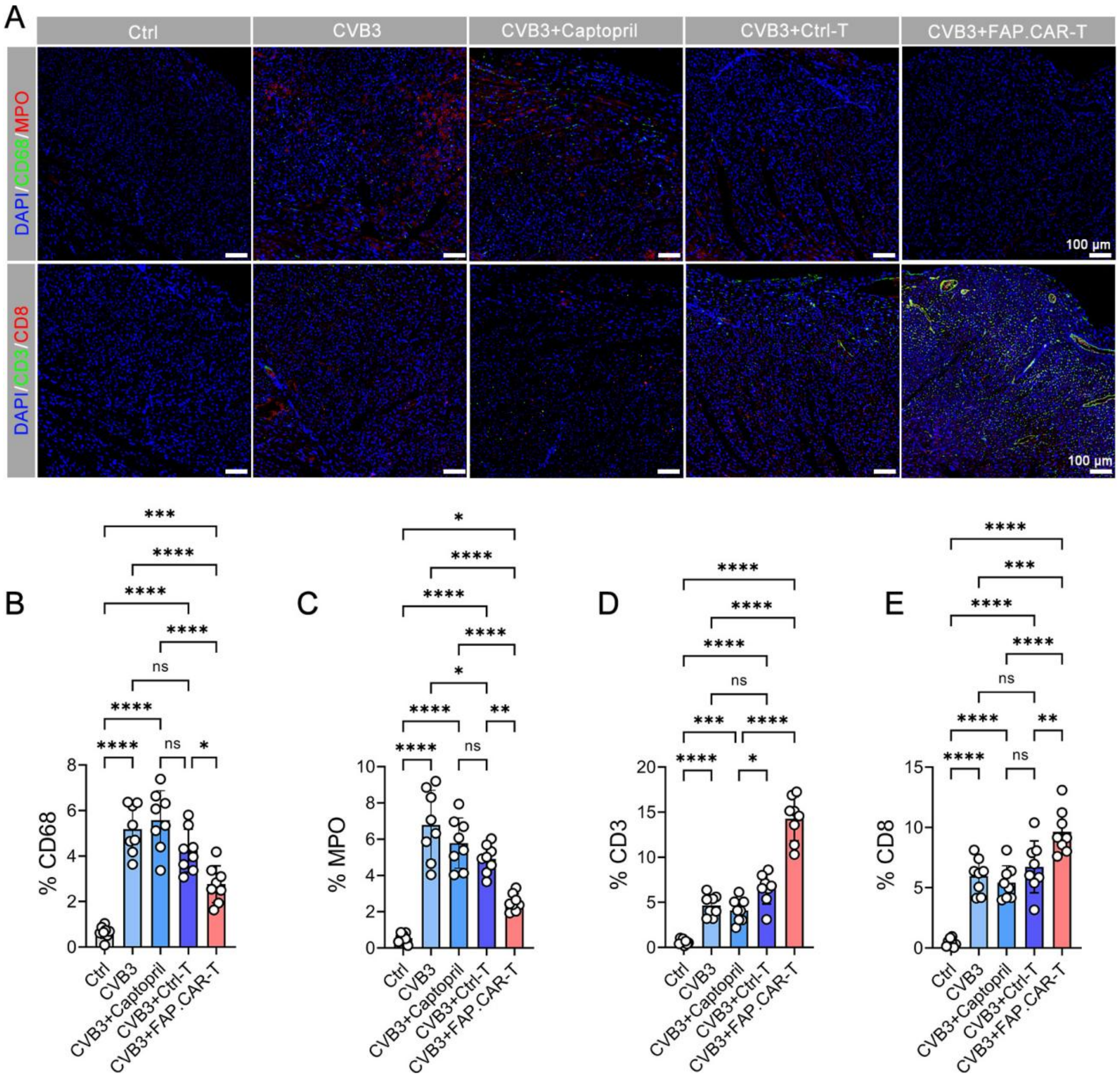

Figure S6. (A) Immunofluorescence staining images and (B-E) statistics of $CD68^+$, MPO^+ cells and $CD3^+$, and $CD8^+$ T cells in heart slices after different treatments in CVB3 mouse model (n = 8 in biologically independent mice per group). Scale bars, 100 μm. Data are represented as mean ± SD. Statistical significance was calculated by ordinary one-way ANOVA, ns = not significance, *P < 0.05, **P < 0.01, ***P < 0.001, and ****P < 0.0001.

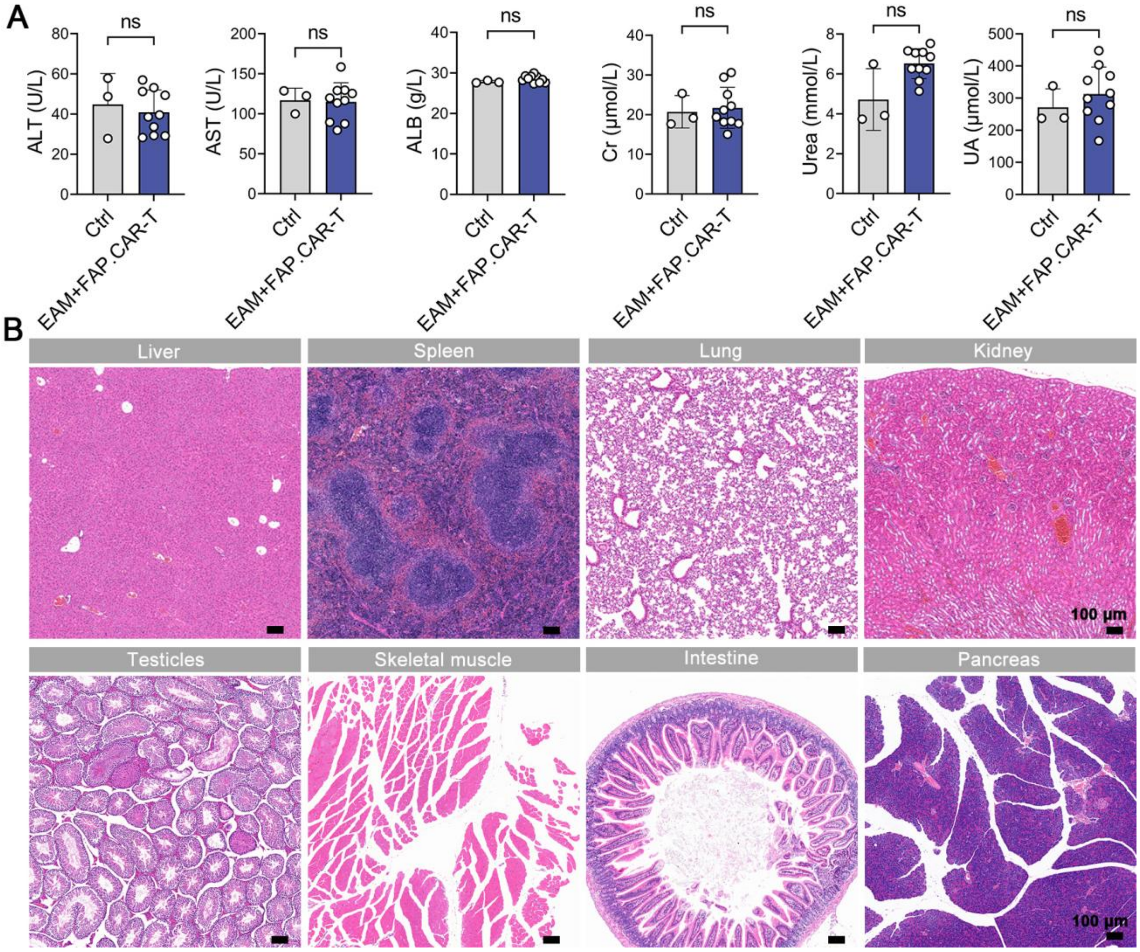

Figure S7. Long term *in vivo* biosafety. (A) Blood biochemistry indexes of mice after treatments (Ctrl: Healthy mice i.v. injection of saline, n = 3; EAM + FAP.CAR-T: EAM-injured mice treated with FAP.CAR-T for 5 months, n = 10). (B) H&E staining of various tissue sections from EAM-injured mice treated with FAP.CAR-T for 5 months (n = 10). Representative images of two independent experiments, showing similar results. Scale bars, 100 μm. Data are represented as mean ± SD. Statistical significance was calculated by two-tailed unpaired Student's t-test in (A), ns = not significance.

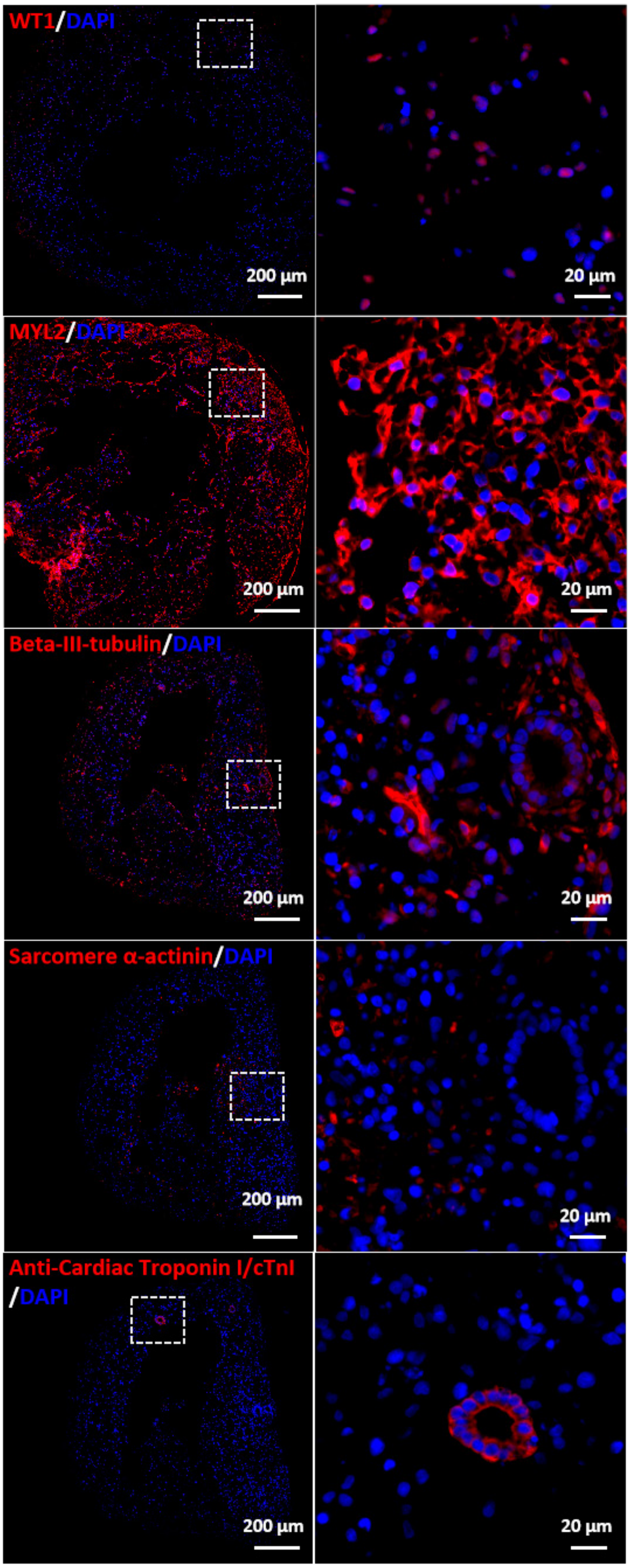

Figure S8. Immunofluorescence staining of human cardiac organoids (hCOs) for DAPI, WT1, MYL2, Beta-III-tubulin, Sarcomere α-actinin, and Cardiac Troponin I/cTnI.

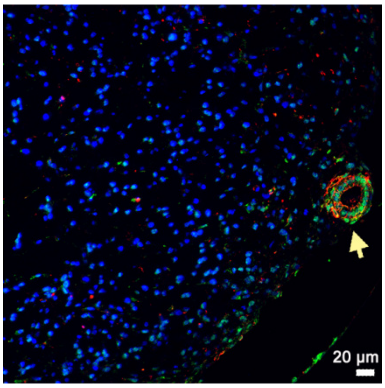

Figure S9. Immunofluorescence staining of human cardiac organoids (hCOs) for DAPI (blue), α-SMA (red), and FAP (green).

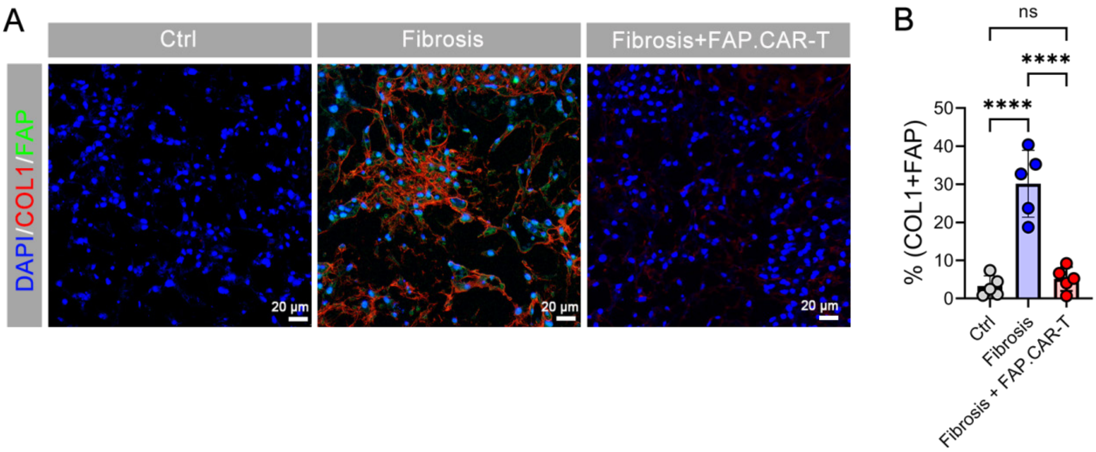

Figure S10. (A) Immunofluorescence staining of hCOs, using DAPI (blue), COL1(red), and FAP (green). (B) Statistics on the co-localization ratio of COL1 and FAP (n=5). Data are represented as mean ± SD. Statistical significance was calculated by ordinary one-way ANOVA, ns = not significance, *P < 0.05, **P < 0.01, ***P < 0.001.

Table S1. Human clinical sample information including 4 MYO in chronic stage and 15 DCM.

Variables	Myocarditis (chronic) (N=4)	DCM (N=15)	*p*-value
Male, n (%)	2 (50)	13 (87)	0.178
Age of onset (years)	45.0 ± 8.3	38.4±11.5	0.303
Age of HTx receiving (years)	51.3 ±8.2	45.7 ± 12.78	0.423
Dyspnoea, n (%)	3 (75)	12 (80)	1.000
Palpitation, n (%)	2 (50)	4 (27)	0.557
Presyncope, n (%)	1 (25)	2 (13)	0.530
Syncope, n (%)	0 (0)	1 (7)	1.000
Abdominal distension, n (%)	0 (0)	4 (27)	0.530
Bilateral oedema, n (%)	1 (25)	7 (47)	0.603
Arrhythmia			
Atrial fibrillation, n (%)	0 (0)	7 (47)	0.245
Ventricular premature contraction, n (%)	1 (25)	2 (13)	0.530
Premature ventricular complexes/24h, n (%)	3 (75)	3 (20)	0.071
Nonsustained ventricular tachycardia, n (%)	3 (75)	3 (20)	0.071
MACE, n (%)	0 (0)	1 (7)	1.000
ICD/CRT/CRT-D, n (%)	1 (25)	0 (0)	0.211

NT-proBNP (fmol/mL)	1622.2 ± 264.0	3823.3 ± 1734.9	<0.001
NYHA, n (%)			
I ~II	2 (50)	0 (0)	0.014
III~IV	2 (50)	15 (100)	
Cardiac ultrasound			
RV dilation, n (%)	2 (50)	4 (27)	0.557
RV wall motion abnormalities, n (%)	2 (50)	1 (7)	0.097
RVEDD (mm)	29.7 ± 8.4	24.8 ± 2.9	
LV dilation, n (%)	3 (75)	15 (100)	0.211
LV wall motion abnormalities, n (%)	4 (100)	15 (100)	-
Interventricular septal thickness (mm)	9 ± 0.82	7.5 ± 0.94	0.011
Thickness of LV (mm)	9.8 ± 0.5	7.6 ± 1.2	0.004
LVEDD (mm)	61.3 ± 13.5	75.8 ± 8.2	0.014
LVEF (%)	35.0 ± 6.7	25.3 ± 5.8	0.010

Variables were expressed as frequency (percent), mean ± SD. Bold italics indicate statistical significance. HTx, heart transplantation. MACE, major adverse cardiovascular events. ICD, implantable cardioverter-defibrillator. CRT, cardiac-resynchronization therapy. CRT-D, cardiac resynchronization therapy defibrillator. NYHA, New York Heart Association. RV, right ventricular. LV, left ventricular. RVEDD, right ventricular end-diastolic diameter. LVEDD, left ventricular end-diastolic diameter. LVEF, left ventricular ejection fraction.

Table S2. Human clinical sample information of varying MYO duration.

Clinical characteristic	<1 year (n=7)	1~6 year (n=7)	6~12 year (n=7)	*p-v*alue
Male, n (%)	3 (42.9)	2 (28.6)	2 (28.6)	0.807
Age of onset, years	28.8 ± 15.7	45.8 ± 10.9	36.4 ± 14.5	0.083
BMI, kg/m2	20.7 ± 2.6	22.6 ± 1.3	19.9 ± 3.7	0.248
Smoking, n (%)	0 (0)	2 (28.6)	0 (0)	0.110
Drink, n (%)	0 (0)	1 (14.3)	1 (14.3)	0.575
Familial disease, n (%)	1 (14.3)	4 (57.1)	4 (57.1)	0.174
Preinfection, n (%)	5 (71.4)	1 (14.3)	1 (14.3)	0.032
Auto-immune disease, n (%)	1 (14.3)	0 (0)	1 (14.3)	0.575
Arrhythmology				
Atrial fibrillation, n (%)	1 (14.3)	0 (0)	4 (57.1)	0.033
NSVT, n (%)	5 (71.4)	3 (42.9)	2 (28.6)	0.263
Premature ventricular contraction, n (%)	4 (57.1)	4 (57.1)	4 (57.1)	1.000
Cardiogenic syncope, n (%)	0 (0)	3 (42.9)	1 (14.3)	0.115

ICD, n (%)	0 (0)	2 (28.6)	2 (28.6)	0.291
NYHA≥III, n (%)	7 (100)	7 (100)	7 (100)	-
Laboratory examination				
NT-proBNP, fmol/ml	7050.1 ± 5891.6	2166.3 ± 1372.2	4253.1 ± 2646.8	0.081
cTnI, ng/mL	0.6 ± 1.6	0.2 ± 0.1	0.1 ± 0.3	0.401
Hemoglobin, g/L	115.9 ± 19.9	135.0 ± 14.5	124.0 ± 22.8	0.207
Sodium, mmol/L	142.6 ± 1.9	141.0 ± 2.5	140.1 ± 2.1	0.132
Creatinine, umol/L	109.7 ± 63.4	83.5 ± 23.0	74.3 ± 20.9	0.268
BUN mmol/L	8.5 ± 3.4	6.8 ± 0.8	5.6 ± 1.7	0.084
Trioxypurine, umol/L	523.4 ± 246.4	420.7 ± 175.1	399.1 ± 175.0	0.483
Albumin（g/L）	41.99 ± 5.9	41.9 ± 3.8	46.0 ± 3.6	0.18
ALT, IU/L	427.8 ± 701.9	31.8 ± 12.9	22.8 ± 14.6	0.131
AST, IU/L	324.2 ± 458.6	27.5 ± 11.8	22.5 ± 5.1	0.076
Total bilirubin, umol/L	47.0 ± 30.1	20.2 ± 11.1	25.6 ± 19.3	0.074
HS-CRP, mg/L	6.7 ± 4.8	4.4 ± 4.6	1.9 ± 1.7	0.105
Triglyceride, mmol/L	1.1 ± 0.4	1.2 ± 0.3	1.5 ± 1.0	0.596
Cardiac ultrasound				

LAD, mm	49.8 ± 4.6	41.7 ± 7.8	44.9 ± 8.4	0.159
LVEDD, mm	61.0 ± 9.3	61.3 ± 14.3	60.0 ± 13.9	0.981
Thickness of LV, mm	8.2 ± 1.9	8.3 ± 1.6	8.4 ± 0.9	0.967
IVS, mm	9.2 ± 0.4	8.5 ± 1.9	8.6 ± 1.3	0.660
LVEF, %	30.2 ± 11.4	32.3 ± 10.9	39.1 ± 12.4	0.359

Variables were expressed as frequency (percent), mean ± SD. Bold italics indicate statistical significance. HTx, heart transplantation. MACE, major adverse cardiovascular events. ICD, implantable cardioverter-defibrillator. CRT, cardiac-resynchronization therapy. CRT-D, cardiac resynchronization therapy defibrillator. NYHA, New York Heart Association. RV, right ventricular. LV, left ventricular. RVEDD, right ventricular end-diastolic diameter. LVEDD, left ventricular end-diastolic diameter. LVEF, left ventricular ejection fraction.

Table S3. Immunohistochemical antibodies list.

Antibody	Item number
anti-FAPα antibody	ab207178 (Abcam)
anti-CD68 antibody	ab283654 (Abcam)
anti-LY6C antibody	ab314120 (Abcam)
anti-MPO antibody	ab208670 (Abcam)
anti-CD3 antibody	ab16669 (Abcam)
anti-CD4 antibody	ab183685 (Abcam)
anti-MYL7 antibody	ab205374 (Abcam)
anti-Myosin Light Chain 2 antibody	ab79935 (Abcam)
anti-Sarcomeric Alpha Actinin antibody	ab137346 (Abcam)
anti-beta lll Tubulin antibody	ab78078 (Abcam)
goat anti-rabbit IgG H&L (Alexa Fluor® 488)	ab150077 (Abcam)
goat anti-rabbit IgG H&L (Alexa Fluor® 594)	ab150080 (Abcam)
and goat anti-rabbit IgG H&L (Alexa Fluor® 647)	ab150079 (Abcam)
anti-CD8 antibody	98941 (Cell Signaling Technology)
anti-WT1 antibody	83535 (Cell Signaling Technology)

Table S4. Flow cytometry antibodies list.

Antibody	Item number
APC-conjugated anti-human CD20 antibody	clone 2H7 (Biolegend)
BV421-conjugated anti-FLAG antibody	clone L5 (Biolegend)
FITC-conjugated anti-mouse CD69 antibody	clone H1.2F3 (Biolegend)
Alexa-Fluor 700-conjugated anti-mouse CD8 antibody	clone 53-6.7 (Biolegend)
PE-Cy5-conjugated anti-mouse CD4 antibody	clone Gk1.5 (Biolegend)
PE-conjugated anti-human NGFR antibody	clone ME20.4 (Biolegend)
Alexa-Fluor 647-conjugated goat-anti-mouse IgG antibody	clone Poly4053 (Biolegend)
PE-conjugated anti-human CD69 antibody	clone FN50 (Biolegend)
PE-conjugated anti-mouse CD3 antibody	clone 500A2 (BD Bioscience)
APC-conjugated anti-human CD3 antibody	clone OKT3 (Thermo eBioscience)

A

Table S5. CSR score of FAP.CAR-T treated mice.

Parameters	Performance (n=10)	CSR score
Fever (>38ºC)	0/10	0
Weight loss	0/10	0
Restlessness	0/10	0
Mental exhaustion	(transient lethargy in 2/10 mice; resolved within 48 h)	0
Fur disarray	0/10	0
